# Antimicrobial Susceptibilities of Oral Isolates of *Abiotrophia* and *Granulicatella* According to the Consensus Guidelines for Fastidious Bacteria

**DOI:** 10.3390/medicines5040129

**Published:** 2018-12-03

**Authors:** Taisei Kanamoto, Shigemi Terakubo, Hideki Nakashima

**Affiliations:** 1 Laboratory of Microbiology, Showa Pharmaceutical University, Machida, Tokyo 194-8543, Japan; 2 Department of Microbiology, St. Marianna University School of Medicine, Kawasaki, Kanagawa 216-8511, Japan; biseibutsu-001@marianna-u.ac.jp (S.T.); nakahide@marianna-u.ac.jp (H.N.)

**Keywords:** nutritionally variant streptococci, antimicrobial susceptibilities, oral microbiota, infective endocarditis

## Abstract

**Background:** The genera *Abiotrophia* and *Granulicatella*, previously known as nutritionally variant streptococci (NVS), are fastidious bacteria requiring vitamin B_6_ analogs for growth. They are members of human normal oral microbiota, and are supposed to be one of the important pathogens for so-called “culture-negative” endocarditis. **Methods:** The type strains and oral isolates identified, by using both phenotypic profiles and the DNA–DNA hybridization method, were examined for susceptibilities to 15 antimicrobial agents including penicillin (benzylpenicillin, ampicillin, amoxicillin, and piperacillin), cephem (cefazolin, ceftazidime, ceftriaxone, and cefaclor), carbapenem (imipenem), aminoglycoside (gentamicin), macrolide (erythromycin), quinolone (ciprofloxacin), tetracycline (minocycline), glycopeptide (vancomycin), and trimethoprim-sulfamethoxazole complex. The minimum inhibitory concentration and susceptibility criterion were determined, according to the consensus guideline from the Clinical and Laboratory Standards Institute. **Results:** Isolates of *Abiotrophia defectiva* were susceptible to ampicillin, amoxicillin ceftriaxone, cefaclor, imipenem, ciprofloxacin, and vancomycin. Isolates of *Granulicatella adiacens* were mostly susceptible to benzylpenicillin, ampicillin, amoxicillin, cefazolin, ceftriaxone, imipenem, minocycline, and vancomycin. The susceptibility profile of *Granulicatella elegans* was similar to that of *G. adiacens*, and the susceptibility rate was higher than that of *G. adiacens*. **Conclusions:** Although *Abiotrophia* and *Granulicatella* strains are hardly distinguishable by their phenotypic characteristics, their susceptibility profiles to the antimicrobial agents were different among the species. Species-related differences in susceptibility of antibiotics should be considered in the clinical treatment for NVS related infections.

## 1. Introduction

The bacteria formerly known as nutritionally variant streptococci (NVS) are characterized by their growth as small satellite colonies supported by helper bacteria such as *Staphylococcus aureus* [1]. The NVS strains require vitamin B_6_ analogs for growth and produce bacteriolytic enzymes, pyrrolidonyl arylamidase and chromophore in common and were supposed to be auxotrophic variants of viridans group streptococci [2]. After several taxonomic alterations, they were finally transferred into two new genera, *Abiotrophia* and *Granulicatella*, on the basis of 16S rRNA gene sequence homology analysis [3,4]. They have been estimated as one of the important pathogens of so-called ‘culture-negative endocarditis’ [2,5,6]; however, because of their fastidiousness in growth, difficulty in identification, and complication in taxonomic position, the clinical importance of these bacteria has been underestimated by clinicians [7].

Although there have been several studies on the antimicrobial susceptibility of NVS, most of the previous studies dealt with a small number of strains, and methods and results were variable [2]. Furthermore, the taxonomic backgrounds of the tested isolates were uncertain. Commercial identification systems, based on the phenotypic characteristics of cultured bacteria, have often misidentified the clinical isolates of *Granulicatella* as *Gemella morbillorum*, and cannot distinguish *Granulicatella adiacens* and *Granulicatella elegans* [8,9,10]. To distinguish the two species of *Granulicatella*, molecular genetic analysis is required [11]. We previously isolated 91 strains of NVS from the human oral microbiota and classified them based on the phenotypic characteristics [9]. Among the oral isolates, 37 isolates confirmed their taxonomic identification by using DNA–DNA hybridization homology analysis, and we reported genetic heterogeneities in genus *Granulicatella* [10].

The Clinical and Laboratory Standards Institute (CLSI) published a laboratory guideline of antimicrobial susceptibility testing of infrequently encountered or fastidious bacteria, not covered in previous CLSI publications [12]. In this study, we determined the minimum inhibitory concentrations (MICs) of the taxonomically confirmed strains of *Abiotrophia* and *Granulicatella*, according to the consensus guideline provided by CLSI.

## 2. Materials and Methods

### 2.1. Bacterial Strains

Seven *Abiotrophia defectiva*, 17 *Granulicatella adiacens*, and six *Granulicatella elegans* (including type strains and oral isolates) were examined (see Table 1 and Table 2). All isolates were identified using the rapid ID32 STREP system (Bio Mérieux SA, Marcy-l’Etoile, France) and DNA-DNA hybridization homology analysis [10]. The reference strains, *A. defectiva* ATCC 49176^T^, NVS-47, and PE7, *G. adiacens* ATCC 49175^T^, and *G. elegans* DSM11693^T^, were from patients with endocarditis or bacteremia [13,14,15], and the other 25 isolates were derived from the oral cavity of healthy volunteers [9]. The strain *Streptococcus pneumoniae* ATCC 49619 was included in the assay to monitor accuracy of the MIC tests. The ATCC strains were obtained from American Type Culture Collection (Manassas, VA, USA), the DSM strain was obtained from Deutsche Sammlung von Mikroorganismen und Zellkulturen GmbH (Braunschweig, Germany), and the other strains were from the stock culture collection in our laboratory.

### 2.2. Antimicrobial Agents

Fifteen antimicrobial agents including penicillin, cephem, carbapenem, aminoglycoside, macrolide, tetracycline, quinolone, glycopeptide, and sulfonamide were used for this study (see Table 3). The following agents were purchased from Wako Pure Chemical Industries, Ltd. (Osaka, Japan): benzylpenicillin, cefazolin, piperacillin, ciprofloxacin, minocycline, and trimethoprim-sulfamethoxazole complex. Ampicillin, ceftazidime, ceftriaxone, cefaclor, gentamicin, erythromycin, and vancomycin were obtained from Sigma Chemical Co. (St. Louis, MO, USA). Amoxicillin was purchased from Fluka Biochemika (Bucks, Switzerland). Imipenem was kindly supplied by the Banyu Pharmaceutical Co., Ltd. (Tokyo, Japan). 

### 2.3. MIC Testing

For preparation of inoculum, tested isolates were cultured anaerobically at 37 °C for 20 to 24 h with Mueller-Hinton broth (MHB; Difco Becton Dickinson and company, Sparks, MD, USA) containing 0.001% pyridoxal hydrochloride (Wako) and the bacterial cell suspensions were adjusted to yield about 5 × 10^5^ CFU/mL. MICs for the *Abiotrophia* and *Granulicatella* strains were determined using the microdilution broth method with MHB containing 2.5% lysed horse blood (Strepto hemo supplement ‘Eiken’, Eiken Chemical Co., Ltd., Tokyo, Japan) and 0.001% pyridoxal hydrochloride, according to the consensus guideline from the CLSI for fastidious bacteria [12]. Briefly, the antimicrobial agents (100 µL/well) were diluted on 96-well round bottom plates (Sumilon, Sumitomo Bakelite Co., Ltd., Tokyo, Japan) in serial two-fold with the supplemented MHB, and 5 µL of the bacterial inoculum was added to each well. The plates were incubated at 35 °C in anaerobic condition for 20 h. The MIC values were defined as the lowest concentrations of antimicrobial agents that completely inhibited the bacterial growth in the microdilution wells, detected by unaided eyes. The strain of *S. pneumoniae* ATCC 49619 was used for quality control testing, and all MIC values for the strain were within the acceptable limits.

### 2.4. Susceptibility Criteria

The MIC values for bacterial isolates to the antimicrobial agents benzylpenicillin, ampicillin, ceftriaxone imipenem, erythromycin, ciprofloxacin, and vancomycin were interpreted into 3 categories: Susceptible, intermediate, and resistant, according to the CLSI guideline for *Abiotrophia* spp. and *Granulicatella* spp.

The MIC values for amoxicillin and piperacillin, and those for cefazolin, ceftazidime, and cefaclor were interpreted using criteria for ampicillin and cephems in the guideline for *Abiotrophia* and *Granulicatella*, respectively [16]. The MIC values for gentamicin and minocycline were interpreted using criteria in the CLSI guideline for *S. aureus* and for *Streptococcus* spp. Viridans group, respectively. The MIC values under 2/38 µg/mL for trimethoprim-sulfamethoxazole were interpreted as susceptible [17].

## 3. Results

The susceptibility percentage of the NVS isolates for 15 antimicrobial agents was summarized in Table 3. Although the phenotypic characteristics of the NVS isolates were similar, the profiles of susceptibility were unique among the species. The NVS isolates were susceptible to ampicillin (96.7%), amoxicillin (100%), imipenem (100%), and vancomycin (96.7%). In addition, *A. defectiva* strains were susceptible to ceftriaxone (100%), cefaclor (85.7%), and ciprofloxacin (100%); and *G. adiacens* strains were susceptible to benzylpenicillin (82.7%), cefazolin (88.2%), ceftriaxone (76.4%), and minocycline (94.1%). The susceptibility profile of *G. elegans* was similar to that of *G. adiacens*, and the susceptibility percentages of *G. elegans* to beta-lactams were higher than that of *G. adiacens*. On the other hand, no NVS strains were susceptible to gentamicin, and 93.3% of the strains were not susceptible to trimethoprim/sulfamethoxazole. Piperacillin susceptibility rate of *A. defectiva* was 0%, while that of *G. adiacens* and *G. elegans* were 52.9% and 100%, respectively. All *A. defectiva* strains were susceptible to ciprofloxacin, but only 17.4% of *Granulicatella* strains were susceptible to it.

Individual MIC values of *A. defectiva* and *Granulicatella* isolates to the antimicrobial agents were shown in Table 1 and Table 2, respectively. Benzylpenicillin-nonsusceptible oral isolates of *A. defectiva* C1-2 and YK-3 were highly resistant to cefazolin and ceftazidime (both MICs = 16 µg/mL) and C1-2 showed additional resistance to cefaclor (MIC = 32 µg/mL), but were susceptible to ceftriaxone (MIC ≤ 1 µg/mL). Oral isolate of *G. adiacens* HHC3 was highly multi-drug resistant to ceftazidime, gentamycin, ciprofloxacin, and minocycline. The benzylpenicillin-nonsusceptible oral isolate of *G. adiacens* P7-4 was resistant to cephems, including ceftazidime, ceftriaxone, and cefaclor (all MICs = 4 µg/mL). Among the NVS isolates, only *G. elegans* DSM11693^T^ was resistant to vancomycin (MIC = 4 µg/mL).

## 4. Discussion

*Abiotrophia* and *Granulicatella* species are very common inhabitants in human normal oral microbiota, in spite of their fastidiousness in growth [9,11,18,19,20], and are significant causative pathogens of endocarditis, bacteremia, and other systemic infections [21,22,23,24]. They often cannot grow on commercial blood agar plates used for the usual clinical examination, and even if they could grow on supplemented culture plates, their colonies are sometimes small, 0.2 to 0.5 mm in diameter [1,25]. Therefore, these fastidious microorganisms have been overlooked in clinical specimens from foci of infective diseases, especially when they are concomitant with easily recovered bacteria (such as *S. aureus*). Based on their phenotypic characteristics, *Abiotrophia* and *Granulicatella* spp. were initially classified as members of genus *Streptococcus*. Although genera *Abiotrophia* and *Granulicatella* were transferred and divided into two groups, based on the 16S rRNA sequence homology analysis, they have been treated as a same bacterial group of NVS in the field of clinical infectious diseases because they have common phenotypic characteristics, such as requiring vitamin B_6_ analogs in growth and producing bacteriolytic enzymes. The human oral cavity is assumed to be a reservoir for the pathogens of many systemic infective diseases, so it is important to examine the antimicrobial susceptibilities of oral bacteria. In this study, we determined MICs of genetically identified seven *Abiotrophia* and 23 *Granulicatella* isolates (including oral isolates), according to the guideline from CLSI. Although NVS species have biochemical and phenotypic properties in common, and are difficult to distinguish without molecular genetical identification methods, the susceptibility profiles to antimicrobial agents were different among the species (Table 3).

Because of their fastidiousness, NVS species often were not recovered from the specimen in the usual clinical examination for infectious diseases caused by these bacteria. When no bacteria are recovered from the specimen of infective diseases, and that happens often, the empiric therapy with broad-spectrum antimicrobial agents (such as carbapenem, macrolide, quinolone, and tetracycline) is selected by the clinicians. As with the antimicrobials tested, all NVS isolates were susceptible to imipenem, and species-related differences were observed with respect to susceptibilities to ciprofloxacin and minocycline. The ciprofloxacin susceptibility rate for *A. defectiva* isolates was 100%, and that for *Granulicatella* isolates was 17.4%. In contrast, the susceptibility rate of minocycline for *Abiotrophia* isolates was 57.1%, and that for *Granulicatella* isolates was 95.7%. Species-related differences in susceptibility of antibiotics should be considered in the empiric therapy for NVS related infections.

In case of infective endocarditis (IE) caused by NVS, a combination of benzylpenicillin and gentamycin has been used for the antibiotic therapy [26,27,28,29]. However, 42.9% of *A. defectiva* isolates were not susceptible to benzylpenicillin and no strains of NVS isolates were susceptible to gentamycin in this study. Aminopenicillins, ampicillin, and amoxicillin showed better susceptible rates than benzylpenicillin and piperacillin. Ceftriaxone and ceftazidime are both third generation cephem, but the susceptible rates were contrary: Only three isolates of *G. elegans* (10.0% of the NVS isolates) were susceptible to ceftazidime. In contrast, 86.6% of the NVS isolates were susceptible to ceftriaxone (Table 3). According to the guidelines for endocarditis treatment by the British Society for Antimicrobial Chemotherapy, vancomycin can be used alone for the NVS IE patients with penicillin allergy [29]. The susceptibility rate of vancomycin for NVS isolates in our study was 96.7%. In the antimicrobial treatment of NVS IE, the recommended initial drugs (the combination of benzylpenicillin and gentamycin) may not be effective, and the regimen of initial drugs should be reconsidered.

Some isolates showed unique susceptibility profiles, for example, *A. defectiva* C1-2 showed multi-drug resistance to piperacillin, cefazolin, ceftazidime, cefaclor, gentamicin, erythromycin, and minocycline, but was susceptible to amoxicillin and ceftriaxone. Some *G. adiacens* isolates, such as HHC3, YTC1, and YTT3, were highly resistant to ceftazidime but susceptible to ceftriaxone, ampicillin, and amoxicillin. *G. adiacens* P7-4 was not susceptible to benzylpenicillin, piperacillin, and cefazolin, and was resistant to ceftriaxone, ceftazidime, and cefaclor, but was susceptible to ampicillin and amoxicillin. In the antimicrobial process, beta-lactams are bound to the penicillin binding protein (PBP) of the bacteria and inhibit their cell wall synthesis. The minor variations of PBP(s) may affect the antimicrobial susceptibilities of these isolates. Further molecular genetic research is needed to determine the mechanism of resistance in the NVS isolates with unique susceptibility profiles.

## Figures and Tables

**Table 1:** MICs (µg/mL) of 15 antibiotics against *Abiotrophia* isolates.

Strains	PEN	AMP	AMX	PIP	CFZ	CAZ	CRO	CEC	IPM	GEN	ERY	CIP	MIN	VAN	SXT
*A. defectiva*															
ATCC49176^T^	0.125	0.016	0.016	1	1	16	1	0.125	0.125	32	0.5	1	0.063	0.25	256/4864
NVS-47	0.125	0.032	0.016	1	2	16	1	0.125	0.125	32	0.5	1	0.032	0.25	256/4864
PE7	0.032	0.016	0.016	4	1	8	1	0.125	0.125	32	0.5	1	0.063	0.25	256/4864
YTS2	0.063	0.016	0.063	1	4	8	0.5	0.5	0.125	32	0.5	1	0.063	0.25	256/4864
C8-3	0.25	0.125	0.063	1	2	8	0.5	0.5	0.125	16	0.25	0.5	4	0.25	0.016/0.3
C1-2	2	0.5	0.25	4	16	16	1	32	0.25	16	128	1	8	0.25	128/2432
YK-3	0.25	0.125	0.125	2	16	16	1	1	0.25	64	4	1	16	0.25	128/2432
range	0.032	0.016	0.016	1	1	8	0.5	0.125	0.125	16	0.25	0.5	0.032		0.016/0.3
	|	|	|	|	|	|	|	|	|	|	|	|	|	0.25	|
	2	0.5	0.25	4	16	16	1	32	0.25	64	128	1	16		256/4864

Type strain and strains NVS-47 and PE7 were derived from blood cultures with endocarditis and the others were oral isolates from healthy volunteers. MIC: minimum inhibitory concentration, PEN: benzylpenicillin, AMP: ampicillin, AMX: amoxicillin, PIP: piperacillin, CFZ: cefazolin, CAZ: ceftazidime, CRO: ceftriaxone, CEC: cefaclor, IPM: imipenem, GEN: gentamicin, ERY: erythromycin, CIP: ciprofloxacin, MIN: minocycline, VAN: vancomycin, SXT: sulfamethoxazole-trimethoprim complex.

**Table 2:** MICs (µg/mL) of 15 antibiotics against *Granulicatella* isolates.

Strains	PEN	AMP	AMX	PIP	CFZ	CAZ	CRO	CEC	IPM	GEN	ERY	CIP	MIN	VAN	SXT
*G. adiacens*															
ATCC49175^T^	0.032	0.032	0.016	0.5	0.125	16	0.25	0.5	0.016	32	0.5	2	0.063	0.5	128/2432
HHC3	0.125	0.063	0.032	1	2	32	0.5	2	0.032	32	0.25	4	8	0.5	64/1216
HHP1	0.063	0.032	0.016	0.25	0.25	4	0.25	1	0.016	32	0.5	2	0.063	0.5	256/4864
P6-1	0.063	0.032	0.016	0.25	0.25	4	0.25	2	0.016	64	0.25	1	0.063	0.5	64/1216
YTC1	0.125	0.063	0.032	0.5	1	32	0.5	2	0.016	16	0.5	2	0.032	0.5	256/4864
S961-2	0.032	0.032	0.016	0.25	0.25	4	2	0.25	0.016	32	0.5	2	0.016	0.5	32/608
S1058-2	0.125	0.032	0.032	0.25	0.25	2	0.25	1	0.016	32	0.25	1	0.032	0.5	32/608
TK-T1	0.032	0.032	0.032	0.25	0.25	4	1	0.5	0.016	32	0.5	2	0.125	0.5	64/1216
HKT1-4	0.25	0.125	0.063	0.5	1	8	1	4	0.032	32	0.5	2	0.125	0.5	32/608
HKT2-2	0.125	0.125	0.063	0.5	1	8	0.25	4	0.032	32	0.125	2	0.016	0.25	32/608
C4-3	0.016	0.008	0.008	0.063	0.125	4	1	0.5	0.016	16	0.25	4	0.016	0.5	256/4864
HKT1-1	0.25	0.125	0.063	0.5	1	16	2	4	0.032	32	0.125	1	0.016	0.25	64/1216
NMP2	0.125	0.125	0.063	0.5	1	4	1	2	0.032	32	0.25	2	0.032	0.5	64/1216
P7-4	0.5	0.25	0.125	0.5	2	4	4	4	0.016	32	0.25	2	0.016	0.5	16/304
S49-2	0.032	0.032	0.032	0.25	0.25	4	8	0.5	0.016	32	0.25	2	0.063	0.5	128/2432
YTT3	0.063	0.032	0.032	0.25	0.25	64	0.25	1	0.032	16	0.25	2	0.125	0.5	64/1216
TK-T2	0.063	0.063	0.063	0.25	0.5	16	1	1	0.016	32	0.5	2	0.125	0.5	256/4864
range	0.016	0.008	0.008	0.063	0.125	2	0.25	0.25	0.016	16	0.125	1	0.016	0.25	16/304
	|	|	|	|	|	|	|	|	|	|	|	|	|	|	|
	0.5	0.25	0.125	1	2	64	8	4	0.032	64	0.5	4	8	0.5	256/4864
*G. elegans*															
DSM11693^T^	0.016	0.063	0.125	0.125	0.125	2	0.5	0.5	0.016	16	8	1	0.25	4	0.5/9.5
NMP3	0.032	0.032	0.016	0.125	0.25	1	0.008	0.5	0.016	16	0.5	2	0.032	0.5	1/19
S1052-1	0.016	0.016	0.016	0.25	0.5	2	0.008	0.5	0.032	16	1	2	0.063	0.5	2/38
YTM1	0.032	0.032	0.016	0.25	0.25	1	0.016	0.5	0.016	16	0.5	4	0.063	0.5	512/9728
HHC5	0.032	0.032	0.016	0.25	0.5	2	0.032	0.5	0.032	8	0.5	2	0.016	0.5	2/38
C9-2	0.063	0.063	0.063	0.25	0.5	1	0.032	2	0.063	16	32	4	2	0.5	1/19
range	0.016	0.016	0.016	0.125	0.125	1	0.08	0.5	0.016	8	0.5	1	0.016	0.5	0.5/9.5
	|	|	|	|	|	|	|	|	|	|	|	|	|	|	|
	0.063	0.63	0.125	0.25	0.5	2	0.5	2	0.63	16	32	4	2	4	512/9728

Type strains were derived from blood cultures with endocarditis and the others were oral isolates from healthy volunteers. MIC: minimum inhibitory concentration, PEN: benzylpenicillin, AMP: ampicillin, AMX: amoxicillin, PIP: piperacillin, CFZ: cefazolin, CAZ: ceftazidime, CRO: ceftriaxone, CEC: cefaclor, IPM: imipenem, GEN: gentamicin, ERY: erythromycin, CIP: ciprofloxacin, MIN: minocycline, VAN: vancomycin, SXT: sulfamethoxazole-trimethoprim complex.

**Table 3:** Percentage of susceptible isolates of *Abiotrophia* and *Granulicatella* against antimicrobial agents.

Antimicrobial Agent	% of Susceptible Isolates		
	*A. defectiva*	*G. adiacens*	*G. elegans*
	(*n* = 7)	(*n* = 17)	(*n* = 6)
Penicillin			
Benzylpenicillin	57.1	82.4	100
Ampicillin	85.7	100	100
Amoxicillin *^a^*	100	100	100
Piperacillin *^a^*	0	52.9	100
Cephem			
Cefazolin *^b^*	28.6	88.2	100
Ceftazidime *^b^*	0	0	50
Ceftriaxone	100	76.4	100
Cefaclor *^b^*	85.7	52.9	83.3
Carbapenem			
Imipenem	100	100	100
Aminoglycoside			
Gentamicin *^c^*	0	0	0
Macrolide			
Erythromycin	14.3	58.8	0
Quinolone			
Ciprofloxacin	100	17.6	16.7
Tetracycline			
Minocycline *^d^*	57.1	94.1	100
Glycopeptide			
Vancomycin	100	100	83.3
Other			
Sulfamethoxazole-trimethoprim *^e^*	14.3	0	16.7

Susceptibilities of the strains to the antimicrobial agents were determined according to the CLSI guideline M45-A2 for *Abiotrophia* spp. and *Granulicatella* spp. Susceptibilities to the antimicrobial agents unlisted in the guideline were determined as below; ^*a*,*b*^ Determined according to the guideline for ampicillin and cephems, respectively; *^c^* Determined according to the CLSI guideline M100-S18 for *S. aureus*; *^d^* Determined according to the CLSI guideline M100-S18 for tetracycline for *Streptococcus* spp. Viridans group; *^e^* Determined by the MIC values under 2/38 µg/mL.
